# Mental Health Literacy: It Is Now Time to Put Knowledge into Practice

**DOI:** 10.3390/ijerph19127030

**Published:** 2022-06-08

**Authors:** Francisco Sampaio, Patrícia Gonçalves, Carlos Sequeira

**Affiliations:** 1 Higher School of Health Fernando Pessoa, Rua Delfim Maia, 334, 4200-253 Porto, Portugal; 2 CINTESIS@RISE—Center for Health Technology and Services Research/Health Research Network from the Lab to the Community, Rua Dr. Plácido da Costa, 4200-450 Porto, Portugal; carlossequeira@esenf.pt; 3 Institute of Health Sciences, Universidade Católica Portuguesa, Rua de Diogo Botelho, 1327, 4169-005 Porto, Portugal; pdgoncalves@porto.ucp.pt; 4 Nursing School of Porto, Rua Dr. António Bernardino de Almeida, 830, 844, 856, 4200-072 Porto, Portugal

## 1. Conceptual Framework and State of the Art

The term ‘mental health literacy’ was first introduced in 1997, and it was defined as ‘knowledge and beliefs about mental disorders which aid their recognition, management or prevention’ [1]. Indeed, mental health literacy is composed of several components, including (a) the ability to recognise specific disorders of types of psychological distress; (b) knowledge and beliefs about risk factors and causes; (c) knowledge and beliefs about self-help interventions; (d) knowledge and beliefs about professional help available; (e) attitudes which facilitate recognition and appropriate help-seeking; and (f) knowledge of how to seek mental health information [2].

Most of the research carried out in this domain has focused on the mental health literacy of adolescents and young adults. Multiple studies have been conducted with results pointing to a similar conclusion: there is a low level of mental health literacy in this population.

For example, a cross-sectional study carried out in the USA in a sample of 1104 adolescents indicated that the participants were more able to recognize depression than social anxiety disorder, and were likely to recommend seeking help, even though less than 50% of those adolescents recognized depression [3]. In another cross-sectional study conducted in Portugal, in a sample of 4938 adolescents and young adults, schizophrenia or psychosis was recognized by only 42.17% and 22.21%, respectively. Additionally, teachers were not seen as a source of help, and there was a reluctance to ask about suicidal feelings [4].

It is important to work on young people’s mental health literacy, as it can enhance positive attitudes around mental health [5]. Given the evidence that adolescents and young adults usually present low levels of mental health literacy, the scientific community has conducted much research on the effectiveness of interventions and programmes that aim to improve it. Although the findings tend to differ from study to study, some interesting conclusions are already available.

Firstly, it seems relevant to state that supporting interventions for improving mental health literacy in adolescents can be categorised into community-based and school-based programmes. Both types use stand-alone education strategies or contact-based groups in addition to education within their programmes. In addition, teaching methods should be interactive and use various media, such as videos, movies and group discussions. Both types of intervention are likely to improve mental health literacy among adolescents [6].

Furthermore, a scoping review to map the structure and context of programmes/interventions to promote mental health literacy among adolescents in the school environment produced some interesting results. This study demonstrated that, despite the positive results of most of the studies analysed regarding the promotion of mental health literacy among adolescents in a school environment, these results were difficult to interpret and compare due to the lack of use of validated instruments and the diversity of the assessment instruments used [7]. In fact, using validated instruments is crucial when assessing mental health literacy [8].

Recently, two systematic reviews with meta-analysis were carried out to determine the effectiveness of mental health literacy interventions in children and adolescents. The results were similar and moderately encouraging: these types of interventions are effective in augmenting mental health knowledge but not in reducing stigma, improving help-seeking behaviours, or reducing social issues, i.e., the willingness to interact with a person with mental illness [9,10].

## 2. A Paradigm Shift

One of the main criticisms associated with the practice and research of the mental health literacy domain is that studies have mainly focused on mental disorders; however, growing evidence demonstrates that supporting positive mental health has long-term benefits [11]. In light of this evidence, a new definition of mental health literacy has been proposed; researchers have suggested adding the component ‘understanding how to obtain and maintain good mental health’ to the former definition [12].

Bjørnsen et al. [11] emphasize that this conceptualization goes beyond previous notions of mental health literacy and the proposed the concept of positive mental health literacy. This concept outlines that mental health literacy has evolved from focusing on mental illness and risk factors to becoming an asset for health that can be strengthened through educational initiatives [12].

In order to consubstantiate this paradigm shift, it is crucial to identify the attributes and characteristics of positive mental health literacy and its theoretical and practical application. Thus, a concept analysis was recently carried out. According to that concept analysis, positive mental health is one component of mental health literacy. The other components include (a) competence in problem-solving and self-actualization; (b) personal satisfaction; (c) autonomy; (d) relatedness and interpersonal relationship skills; (e) self-control; and (f) a prosocial attitude [13].

As this is a recent construct or, at least, a recent way of looking at mental health literacy, there are still few studies assessing positive mental health literacy. Nevertheless, a study published in 2019 sought to examine the relationship between positive mental health and mental well-being in a sample of 1888 adolescents aged 15–21 years. The results were quite promising: the regression model accounted for 41% of the variance in adolescents’ mental well-being, and positive mental health literacy was a significant explanatory variable of mental well-being. These findings highlight the need to include positive mental health literacy as an integral component of the mental health services provided in schools for adolescents [14].

The abovementioned study looked at positive mental health literacy as a resource that may lead to an increase in mental well-being. Thus, the results of this study are a valuable resource. Nonetheless, it is also important to investigate positive mental health literacy as an outcome. Notably, an assessment tool that enables the assessment of positive mental health literacy has already been developed and validated among Norwegian adolescents [11]. Therefore, it is critical to replicate this method in samples from different countries, as this is likely to lead to effective ways of assessing the efficacy and/or effectiveness of psychoeducational interventions designed to increase positive mental health literacy.

## 3. Future Challenges

There is no doubt that mental health literacy is currently a ’hot topic’ in mental health research. However, it is now time for a paradigm shift in which mental health literacy is viewed not only as knowledge about mental disorders and their risk factors, but also as a construct that promotes positive mental health.

Nonetheless, there are future challenges regarding research in the mental health literacy domain. Most of the studies on this topic focus on children, adolescents, and/or young adults [15]. Consequently, there is a substantial lack of information about mental health literacy in adults, the elderly, and people with specific conditions, such as people with mental disorders, people with terminal illness, and informal caregivers. Therefore, it is of paramount importance to examine mental health literacy in these populations and develop and evaluate the efficacy of the psychoeducational interventions/programmes that aim to improve it.

There is an important gap between mental health research and mental health practice [16], which is also true in the mental health literacy domain. There are several studies about mental health literacy, some providing high levels of evidence. However, there are a lack of psychoeducational interventions/programmes effectively implemented in clinical practice. School nurses could be an important resource to help improve students’ mental health literacy [14]; however, not all school nurses present a high level of mental health literacy themselves. For example, a study conducted in the United Arab Emirates pinpointed that less than 50% of the respondents (339 school nurses) correctly identified the mental disorders presented in three vignettes of fictional characters, and the accurate identification of evidence-based interventions was also limited [17].

Considering the low level of mental health literacy presented by some school nurses, mental health nurses could potentially bridge that gap. Because they are mental health specialists and with inherent knowledge about mental health issues (not only about mental disorders but also about how to promote mental health), these professionals could provide mental health education for children, adolescents, and/or young adults in schools. Moreover, they could provide mental health education for the teachers so that they can proactively identify mental health problems, constitute a mental health first aid resource for students, and/or refer them to mental health specialists if needed.

## Data Availability

Not applicable.
